# The Impact of COVID-19 Pandemic First Wave on Healthcare Workers: A New Perspective from Qualifying PTSD Criterion A to Assessing Post-Traumatic Growth

**DOI:** 10.3390/jcm12051862

**Published:** 2023-02-27

**Authors:** Camilla Gesi, Giovanna Cirnigliaro, Francesco Achilli, Matteo Cerioli, Rita Cafaro, Maria Boscacci, Bernardo Dell’Osso

**Affiliations:** 1 Department of Biomedical and Clinical Sciences Luigi Sacco, Department of Psychiatry, ASST Fatebenefratelli-Sacco, University of Milan, 20157 Milan, Italy; 2 Department of Psychiatry and Behavioral Sciences, Bipolar Disorders Clinic, Stanford Medical School, Stanford University, Stanford, CA 94305, USA; 3 CRC “Aldo Ravelli” for Neurotechnology & Experimental Brain Therapeutics, University of Milan, 20122 Milan, Italy; 4 Centro per lo Studio dei Meccanismi Molecolari alla Base delle Patologie Neuro-Psico-Geriatriche, University of Milan, 20122 Milan, Italy

**Keywords:** COVID-19 pandemic, post-traumatic stress disorder, post-traumatic growth, PTSD criterion A, healthcare workers, psychological impact

## Abstract

Post-traumatic growth (PTG) and specific traumatic events have been poorly explored in the literature focusing on post-traumatic stress disorder (PTSD) among healthcare workers (HWs) tackling the COVID-19 pandemic. In a large sample of Italian HWs, we investigated the kinds of traumatic events and whether PTG affects the risk of PTSD, along with its prevalence and features, during the first COVID-19 wave. COVID-19-related stressful events, Impact of Event Scale-Revised (IES-R) and PTG Inventory-Short Form (PTGI-SF) scores were collected through an online survey. Out of 930 HWs included in the final sample, 257 (27.6%) received a provisional PTSD diagnosis based on IES-R scores. Events referring to the overall pandemic (40%) and to a threat to a family member (31%) were reported as the most stressful events. Female sex, previous mental disorders, job seniority, unusual exposure to sufferance and experiencing a threat to one’s family significantly increased the provisional PTSD diagnosis’ risk, while being a physician, the availability of personal protective equipment and moderate/greater scores on the PTGI-SF spiritual change domain were found to be protective factors.

## 1. Introduction

COVID-19 has caused a massive death toll around the world and delivered various challenges to the mental wellbeing of many people. The first Italian hotbed of SARS-CoV-2 started in February 2020; soon after, the epidemic spread across the entire country, leading to Italian hospitals rapidly running out of beds, workforce, and supplies. Besides the risk of infection, Healthcare Workers (HWs) had to tackle overflowing hospitals and extenuating work shifts, while being exposed to suffering and deaths on an unprecedented scale. Despite professional training and expertise, HWs’ likelihood of experiencing traumatic events sharply increased during this phase of the pandemic. 

Several online surveys have been undertaken during the first wave of the pandemic to evaluate the impact of COVID-19 on HWs’ psychological wellbeing, with most studies focusing on anxiety, sleep, depression, and especially post-traumatic symptoms [1]. However, all previous studies investigating post-traumatic symptoms referred to the whole pandemic as a traumatic event, while none explored whether specific events occurring during the pandemic met the Post-Traumatic Stress Disorder (PTSD) criterion A for qualifying traumatic event [2]. On the other hand, most previous studies have not considered possible positive outcomes of COVID-19, their correlates, and their relationship with PTSD symptoms’ development. It has been suggested that people enduring traumatic or challenging events may not only develop PTSD symptoms but also experience positive changes, referred to as post-traumatic growth (PTG), and entailing different dimensions of one’s life (such as an increased appreciation for life, more meaningful interpersonal relationships, an increased sense of personal strength, changes in one’s priorities, a richer spiritual life) [3,4]. To the best of our knowledge, the few studies considering the role of PTG during the COVID-19 pandemic have only been conducted on specific HWs populations (e.g., nurses) [5,6,7], and none have been conducted in Italy so far.

In light of the great importance of elucidating factors leading to/protecting against PTSD symptoms in such a valuable population, the aim of this study was to assess the prevalence of PTSD and its correlates in a large sample of Italian HWs during the first COVID-19 wave. The question of which specific COVID-19-related events were most distressing and whether PTG played a role against the development of PTSD symptoms was also investigated.

## 2. Materials and Methods

### 2.1. Study Design and Participants

We conducted a cross-sectional study assessing socio-demographic characteristics as well as the Impact of Event Scale-Revised (IES-R) and the Post-Traumatic-Growth-Inventory-Short-Form (PTGI-SF) scores in a sample of Italian HWs during the first wave of the COVID-19 pandemic. Data were collected through an online survey between 4 April and 13 May 2020, the late phase of the first stay-at-home order during the first wave of COVID-19 in Italy. An invitation to take part in the survey was addressed to HWs actively working in Italy, with no exclusion in relation to diagnostic, rehabilitation, and technical personnel, and it was vehiculated through healthcare institutions, associations, and social networks groups. Participants gave their informed consent to participate in the study and to have their data used for research purposes. Their answers were collected anonymously. The study was conducted in accordance with the principles stated in the Declaration of Helsinki, and study procedures were approved by the Department of Psychiatry of the ASST Fatebenefratelli-Sacco of Milan as a relevant institution review board for low-risk studies (code: dsm 12-20).

### 2.2. Assessments

The survey consisted of three sections. The first section included questions about socio-demographic variables and changes that may have occurred within the family or workplace due to COVID-19. The second section was introduced by a gate question: “Would you say you have been through a very stressful/traumatic experience because of the COVID-19 pandemic?”. Upon answering ‘yes’, participants were asked to briefly describe the event and to fill the Impact of Event Scale-Revised (IES-R). Based on independent qualitative analyses by two researchers of the events described, answers were then clustered into four categories: Threat to family, Threat to self, Workload and organizational problems, and Overall pandemic. In the third section of the survey, participants were offered to fill the Post Traumatic Growth Inventory-Short Form (PTGI-SF).

### 2.3. Impact of Event Scale-Revised (IES-R)

The IES-R was used to assess PTSD symptoms [8]. The scale consists of 22 items, scoring on a five-point Likert scale, and divided in three subscales (respectively investigating intrusion, avoidance, and hyperarousal symptoms in the last 15 days). A score of 33 or higher on the IES-R is suggestive of a provisional diagnosis of PTSD, while a score of 24 or higher has been used to indicate partial PTSD [9]. Both the original and the Italian IES-R version have been extensively validated and have shown good psychometric properties [10]. High levels of internal consistency have been reported for each subscale of the original IES-R version (intrusion: Cronbach’s alpha = 0.94, avoidance: Cronbach’s alpha = 0.87, hyperarousal: Cronbach’s alpha = 0.91) [11] and the Italian IES-R version (intrusion: Cronbach’s alpha = 0.78, avoidance: Cronbach’s alpha = 0.72, hyperarousal: Cronbach’s alpha = 0.83) [10]. Its usability in assessing PTSD related to the COVID-19 pandemic has also been validated [12]. In our sample, it showed a good reliability (Cronbach’s alpha = 0.93).

### 2.4. Post-Traumatic Growth Inventory-Short Form (PTGI-SF)

Post-traumatic growth was investigated through the Italian version of the PTGI-SF [13], aimed to assess post-traumatic growth across five dimensions: appreciation of life, relating to others, personal strength, spiritual change, and new possibilities. The 10 items are coded on a 6-point Likert scale, with greater scores indicating higher levels of post-traumatic growth [3]. The Italian PTGI-SF was derived from the Italian full-length Post-Traumatic Growth Inventory (PTGI), showing a good internal consistency (Cronbach’s alpha = 0.93) [13]. The five dimensions of the PTGI-SF showed an acceptable-to-good internal consistency (Cronbach’s alpha values ranging from 0.70 to 0.82) and high correlation with the original PTGI factor scores (r values ranging from 0.90 to 1.00) [13].

In our sample, the PTGI-SF showed good psychometric properties (Cronbach’s alpha = 0.874).

### 2.5. Statistical Analysis

Comparisons of IES-R and PTGI-SF scores between subjects showing different socio-demographic and work-related characteristics were conducted using the Student *t*-test or the one-way ANOVA where appropriate, as the two scales’ scores showed a normal distribution in our sample (Shapiro–Wilk normality test, IES-R *p* < 0.001; PTGI-SF *p* = 0.002). Post-hoc comparisons were conducted using the Tukey test. The percentages of participants meeting a provisional full or partial PTSD diagnosis were computed. Binary logistic regression analysis was conducted to investigate the contribution of each socio-demographic, clinical, and occupational factor to the provisional PTSD diagnosis.

## 3. Results

A total of 1044 subjects completed the survey. As the survey was vehiculated with the assistance of healthcare institutions, associations and social networks, the response rate could not be calculated. After data cleaning, 114 subjects were excluded for incomplete/inconsistent data or duplicated records. The final sample thus included 930 HWs. The mean (SD) age was 45.1 (11.8) years, 592 (63.7%) participants were females, 441 (47.4%) were from Lombardy, and 489 (52.6%) were from other Italian regions. A total of 723 (77.7%) were physicians, 104 (11.2%) were nurses, and 103 (11.1%) were a mixed group mostly composed of midwives, rehabilitation personnel, and laboratory technicians.

### 3.1. Post-Traumatic and Post-Traumatic Growth Features

**Figure 1:**
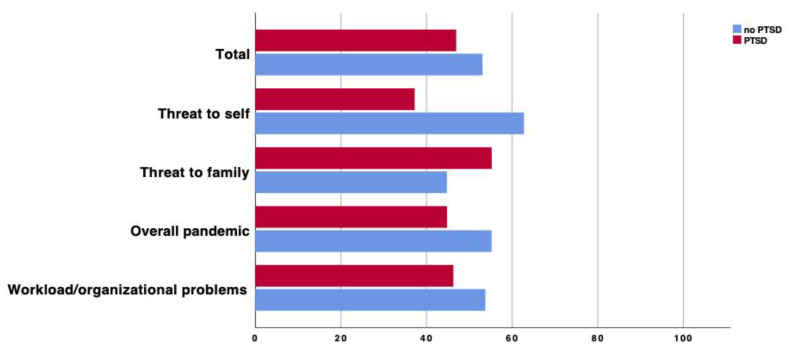
Prevalence of putative PTSD among 554 IES-R respondents by type of traumatic event.

A total of 554 HWs (59.5% of the total sample) answered ‘yes’ to the gate question about a COVID-19-related traumatic event and filled the IES-R. The mean IES-R score was 32.0 ± 16.5. A total of 356 HWs (64.9% of IES-R respondents) screened positive for at least putative partial PTSD (IES-R score ≥ 24), and 257 (46.5% of IES-R respondents, 27.6% of the total sample) did so for full putative PTSD (IES-R ≥ 33). Based on the qualitative analysis of pandemic-related events indicated as most stressful, 94 (17.0%) of the IES-R respondents described an episode falling under the category Threat to self (such as close, unprotected contacts with COVID-19 confirmed cases or being diagnosed with COVID-19); 172 (31.1%) answers belonged to the category Threat to family (e.g., COVID-19 suspected symptoms among relatives or the fear of vehiculating the virus within the family); 221 (40.0%) indicated Overall pandemic (including a great exposure to suffering patients, difficulties in the management of many unstable patients or a difficulty dealing with relatives’ phone calls and requests) as the most stressful event; and 67 (12.1%) indicated an overwhelming workload and organizational problems (such as patients’ overflow, a lack of or vague directions from hospital administrators or poor communication within the team). The percentages of subjects exceeding the IES-R cut-off score for a putative full PTSD diagnosis across different trauma groups are shown in Figure 1.

Bivariate analyses showed that the IES-R mean score was higher among participants with living parents (*p* = 0.05), minor children (*p* = 0.05), or among those who temporarily got separated from cohabiting relatives (*p* < 0.001) due to the pandemic. HWs who were relocated to other units (*p* = 0.04), experienced an increased workload (*p* = 0.01) or were exposed to unusual suffering (*p* < 0.001) reported higher IES-R scores as well. The mean PTGI-SF score was 23.5 ± 9.7, with the highest sub-component mean scores being in the domains of appreciation of life (5.4 ± 2.2) and personal strength (5.4 ± 2.4) and the lowest scores being in the domain of spiritual change (3.3 ± 2.8). Significantly higher PTGI-SF scores were reported by nurses compared with physicians (*p* = 0.04), HWs outside Lombardy (*p* = 0.002) and those who completed the survey before 4th May (*p* = 0.005). No differences in PTGI-SF scores were found based on the type of reported event or other work-related variables. The complete data on IES-R and PTGI-SF scores by participants’ socio-demographic, work-related and clinical characteristics are available in Supplementary Materials.

### 3.2. Predictors of PTSD

Table 1 shows the results of a binary logistic regression analysis conducted using the putative PTSD diagnosis obtained from the IES-R as the dependent variable and several variables, including PTGI-SF scores, as the predictors. Female sex (OR = 1.62; 95% CI = 1.04–2.52), previous mental disorders (OR = 1.6; 95% CI = 1.03–2.47), less than 15 years of job seniority (OR = 1.87; 95% CI = 0.99–3.52), unusual exposure to suffering (OR = 3.16; 95% CI = 2.02–4.92) and exposure to a traumatic event implying a threat to family (compared to a threat to self) (OR = 2.07; 95% CI = 1.11–3.83) significantly increased the risk of receiving a putative diagnosis of PTSD, while being a physician (compared to other professional figures) (OR = 0.35; 95% CI = 0.715–0.84), the availability of personal protective equipment (PPE; OR = 0.53; 95% CI = 0.35–0.81) and moderate or greater scores on the PTGI-SF spiritual change domain (OR = 0.544; 95% CI = 0.346–0.832), were found to be protective factors in relation to the PTSD putative diagnosis.

## 4. Discussion

The present cross-sectional study conducted on a sample of Italian HWs during the first pandemic phase sought to investigate the prevalence of PTSD and its associated features. Moreover, we investigated which specific COVID-19-related events were described as being most traumatic and whether positive factors (i.e., post-traumatic growth) played a role against the development of PTSD symptoms.

### 4.1. Type of Trauma, PTSD Prevalence and Post-Traumatic Growth Features

Almost 60% of respondents to the survey indicated that they experienced some sort of traumatic event during the pandemic and were therefore offered to fill in the IES-R. About half of them reached the cut-off for a putative diagnosis of PTSD, corresponding to 27% of the overall sample, a percentage falling toward the lower end of prevalence estimates provided by previous studies [9,14,15,16,17,18,19]. Most IES-R completers (40%) described the overall pandemic as a traumatic event. Notwithstanding the fact that most previous studies assessed PTSD symptoms related to the pandemic as a whole, we may argue that HWs in this group experienced very different levels of traumatic exposure, failing to endorse PTSD criterion A (American Psychiatric Association, 2013) in some instances. Events threatening family or threatening the self were reported by 30% and 17% of IES-R respondents, respectively. Such events mostly concerned a suspected or confirmed COVID-19 infection of oneself or one’s closest ones, therefore meeting DSM-5 criterion A for PTSD (direct or family exposure to threatened death). Interestingly, the threat to a family member was significantly associated with a higher overall IES-R score compared to the threat to self and was found to be a significant predictor of PTSD in the multivariate analysis. These data show the central role of family bonds, which is corroborated by the IES-R scores being significantly higher in those with living parents, minor children, and those who had to separate from their family due to the virus [20]. These data also indicate the potential usefulness of providing HWs with special housing and/or accommodation, as implemented by some governments, and highlights the need to include family members into measures of work-related risk management for HWs [21]. A total of 12% of participants who filled the IES-R identified an overwhelming workload and organizational problems as traumatic experiences. While PTSD criterion A is not clearly met by HWs indicating this type of event, 31 subjects (46.3%) in this group exceeded the cut-off of 33 for receiving a putative PTSD diagnosis in proceeding with the IES-R. This may point out, on one hand, that significant levels of post-traumatic symptoms may happen to arise from low-magnitude events, falling outside the boundaries of DSM-5 criterion A. On the other hand, it may suggest caution in generalizing data provided by studies assessing PTSD symptoms irrespectively of the type of trauma to clinician-diagnosed populations [22]. Post-traumatic growth was especially endorsed in the dimensions of appreciation of life and personal strength, while the dimension with the lowest mean scores, spiritual growth, was the only one associated with significantly lower odds for PTSD in the multivariate analysis (see below). Interestingly, some factors were associated with both higher PTSD symptoms and higher post-traumatic growth in the bivariate analyses (i.e., female sex), confirming that negative and positive effects of trauma can coexist simultaneously [23]. Somewhat unexpectedly, no relationship was found between different types of referred trauma and PTG dimensions. One possible explanation may be that subjects were allowed to indicate only one traumatic event, while the dimensions assessed by the IES-R and PTGI-SF questionnaires might subdue the cumulative effect of multiple events experienced throughout the whole pandemic.

### 4.2. Predictors of PTSD

Our data showed that PTSD symptoms during the first wave of COVID-19 were associated with a constellation of clinical, demographic, and work-related variables.

As for clinical variables, we found that the odds for a putative PTSD diagnosis were higher among HWs with a previous mental disorder, confirming a solid finding across several lines of PTSD literature [24,25,26,27], yet shown for the first time in a sample of HWs during COVID-19. Experiencing an event which implied a threat to family stood out among other types of events as a significant predictor of PTSD symptoms, according to the criterion A1 of PTSD (American Psychiatric Association, 2013), as already discussed above. Concerning work-related variables, being a physician was associated with lower odds for PTSD, in agreement with previous data reporting a higher risk among nurses compared to other professional figures including physicians [1,18,28,29], although some studies reported no differences between nurses and medical staff [16]. Concurrently, no significant difference was found between HWs working in frontline units versus those working in other medical units. This finding is in contrast with previous studies showing higher perceived stress among frontline workers compared to others HWs [29,30,31,32]. On the other hand, we found that unusual exposure to suffering was a risk factor for PTSD development, depicting the hypothesis that the exposure to high levels of work-related stress without adequate training—as may happen to no frontline HWs—might clear the way for PTSD [33]. Further, female sex was found to be a risk factor for the development of PTSD, consistent with most previous literature [28,34].

We found two variables that stood out as protective factors for PTSD diagnosis. The first one was the availability of personal protective equipment (PPE), which was dramatically lacking during the first wave of COVID-19 in Italian Hospitals [34,35,36]. Secondly, spiritual growth, while being the PTGI-SF dimension with the lowest mean score, was found to reduce the odds for PTSD. This finding aligns with a recent study showing the lowest PTGI-SF domain endorsement in spiritual growth among HWs tackling the first wave of COVID-19 at an urban hospital in NYC [5]. Interestingly, the same study found an association between spiritual growth endorsement and lower odds of screening positive for pandemic-related PTSD symptoms 7 months after the first wave, suggesting that the positive effect of PTG may last across time or even arise at some point during the process of adjusting to trauma. From this perspective, as PTG and PTSD are not to be considered mutually exclusive, PTG may be profitably used in the treatment of PTSD symptoms by prompting reflective processing of the event and positive changes throughout healing [37,38]. Unfortunately, due to the cross-sectional design of the study, it was not possible to determine whether the onset of PTSD symptoms preceded or followed that of PTG. The study has some further limitations, first and foremost the use of self-reporting instruments. Secondly, subjects were allowed to describe only one traumatic event, while multiple events with different magnitudes arguably contributed to HWs’ traumatic load during the pandemic. Moreover, the lack of follow-up data about PTG and PTSD prevented us from drawing a thorough picture about the relationship between PTSD symptoms and PTG dimensions.

### 4.3. Modifiable Factors in the Management of the Pandemic

Among variables that we found to be relevant to PTSD development, modifiable factors play an important role in helping health authorities and HWs to tackle and adjust to events such as a pandemic. Given the importance of family safety found in our study, special housing should be available for HWs with cohabiting, vulnerable family members. Moreover, the availability of PPE and other protection measures should be prioritized in order to build a safe work environment. Lastly, additional training should be provided to all HWs involved in big emergencies, and psychological/psychiatric support should be routinely offered.

## 5. Conclusions

The present study confirmed that a putative PTSD diagnosis was frequently reported among HWs facing the first COVID-19 pandemic wave and identified factors that may significantly impact PTSD development, including factors that may play a protective role, such as some dimensions entailed in PTG. We also contributed to elucidating which events were perceived as being most distressing by HWs and highlighted the relevance of events threating family safety to the development of PTSD. Longitudinal studies are warranted to investigate the consistency of our findings across the later phases of the pandemic, especially regarding PTG.

## Figures and Tables

**Table 1:** Binary logistic regression analysis with provisional PTSD diagnosis as dependent variable.

	B (SE)	Wald	df	*p*	OR	OR 95% CI	
						Lower	Upper
Sex [Male]	0.48 (0.23)	4.59	1	**0.03**	1.62	1.04	2.52
Age [Older than 40]	−0.46 (0.32)	2.09	1	0.15	0.63	0.34	1.18
Region [Lombardy]	0.21 (0.25)	0.68	1	0.41	0.801	0.75	2.01
Date of completion [Before May 4th]	−0.24 (0.28)	0.76	1	0.38	0.79	0.46	1.35
Professional Role [Other]		6.27	2	**0.04**			
Nurse	−0.538 (0.36)	1.11	1	0.29	0.69	0.34	1.39
Physician	−1.05 (0.45)	5.57	1	**0.02**	0.35	0.715	0.84
Job seniority [more than 15 years]	0.63 (0.32)	3.77	1	**0.05**	1.87	0.99	3.52
Previous mental disorders	0.47 (0.22)	4.40	1	**0.04**	1.60	1.03	2.47
Workplace [Frontline]		3.25	4	0.52			
Medicine	0.65 (0.56)	1.32	1	0.25	1.91	0.63	5.78
Surgery	−0.01 (0.49)	0.01	1	0.99	1.00	0.37	2.67
Territorial medicine	0.01 (0.50)	0.01	1	0.98	1.01	0.39	2.63
Services/not specified	0.16 (0.50)	0.10	1	0.75	1.17	0.44	3.10
Availability of PPE ^1^	−0.63 (0.22)	8.44	1	**0.01**	0.53	0.35	0.81
Type of trauma [Threat to self]		6.69	3	0.08			
Work-related issues	0.566 (0.38)	2.17	1	0.14	1.76	0.83	3.74
Overall pandemic	0.26 (0.30)	0.77	1	0.38	1.30	0.72	2.35
Threat to family	0.73 (0.32)	5.29	1	**0.02**	2.07	1.11	3.83
Infected (self)	0.38 (0.34)	1.28	1	0.26	1.46	0.76	2.83
Infected family members	0.139 (0.23)	0.37	1	0.55	1.15	0.73	1.80
Deceased family members	0.124 (0.23)	0.29	1	0.59	1.13	0.72	1.79
Separation from cohabiting family	−46 (0.28)	2.78	1	0.10	0.63	0.37	1.09
Separation from non-cohabiting family	−0.20 (0.41)	0.23	1	0.63	0.82	0.37	1.84
Minor children	−0.15 (0.210)	0.49	1	0.48	0.86	0.57	1.30
Living parents	0.04 (0.27)	0.03	1	0.88	1.04	0.61	1.78
Change in usual tasks	0.09 (0.26)	0.12	1	0.73	0.91	0.54	1.84
Relocation to other units	0.01 (0.31)	0.01	1	0.99	0.99	0.54	1.82
Relocation to COVID-19 units	0.41 (0.27)	2.37	1	0.123	1.50	0.90	2.53
Unusual exposure to suffering	1.149 (0.23)	25.54	1	**<0.01**	3.16	2.02	4.92
PTG-SF ^2^ relating to others	−0.14 (0.25)	0.031	1	0.58	0.87	0.53	1.42
PTG-SF ^2^ spiritual change	−0.62 (0.23)	7.75	1	**<0.01**	0.544	0.346	0.832
PTG-SF ^2^ appreciation of life	−0.33 (0.30)	1.23	1	0.27	0.72	0.40	1.29
PTG-SF ^2^ new possibilities	0.34 (0.27)	1.64	1	0.20	1.41	0.83	2.39
PTG-SF ^2^ personal strength	0.48 (0.28)	2.94	1	0.09	1.63	0.93	2.83

^1^ Personal Protective Equipment; ^2^ Post Traumatic Growth Inventory-Short Form.

## Data Availability

The data underlying this article will be shared on reasonable request to the corresponding author.

## Supplementary Materials

The following supporting information can be downloaded at: https://www.mdpi.com/article/10.3390/jcm12051862/s1, Table S1: IES-R and PTGI-SF scores by participant characteristics (n = 554).
