# Supplementary material for: The Impact of COVID-19 Pandemic First Wave on Healthcare Workers: A New Perspective from Qualifying PTSD Criterion A to Assessing Post-Traumatic Growth
Source: J Clin Med. 2023 Feb 27;12(5):1862. doi: 10.3390/jcm12051862 (PMC10003652; doi:10.3390/jcm12051862)
Supplement: Supplementary file 1 [file jcm-12-01862-s001.zip › jcm-2150057-supplementary.pdf]

Table S1: IES-R and PTGI-SF scores by participant characteristics (n=554).

	IES-R[1] (n=554)			PTGI-SF[2] (n=548)		
	**Mean±SD**	**t/F**	**P value**	**Mean±SD**	**t/F**	**P value**
**Age**						
Younger than 40	32.4±16.6	.44	.66	22.7±10.0	-1.51	.13
Older than 40	31.8±16.4			24.0±9.4		
**Sex**						
Male	27.4±17.1	4.51	**<.001**	20.7±9.7	4.78	**<.001**
Female	34.2±15.7			24.8±9.3		
**Minor children**						
No	31.0±16.2	-1.93	**.05**	23.1±9.5	-1.16	.25
Yes	33.7±16.8			24.1±9.9		
**Living parents**						
No	29.4±16.1	-1.99	**.05**	23.8±9.3	.34	.73
Yes	32.8±16.5			23.4±9.8		
**Region**						
Lombardia	31.2±16.6	1.18	.24	22.2±10.2	3.06	**<.01**
Other regions	32.9±16.3			24.8±8.9		
**Date of completion**						
Before May 4, 2020	32.8±16.5	-1,72	.09	24.2±9.4	-2.81	**<.01**
May 4, 2020 or later	30.0±16.4			21.6±10.1		
**Professional role**						
Physician	31.9±16.5			23.2±9.5		
Nurse	30.5±16.4	1.02	.36	26.3±10.5	3.22	**.04***
Other	35.0±16.6			22.6±9.2		
**Job seniority**						
up to 15 years	33.4±16.7	1.90	.06	23.0±10.0	-1.18	.24
more than 15 years	30.7±16.2			24.0±9.2		
**Previous mental disorders**						
No	30.4±16.6	-3.60	**<.001**	23.6±9.5	.53	.60
Yes	35.8±15.5			23.1±10.1		
**Workplace**						
Frontline	28.0±17.5			23.0±9.7		
Medicine	33.6±16.7	2.22	.07	22.8±9.8	.43	.79
Surgery	29.5±16.8			23.7±9.4		
Territorial Medicine	34.5±16.4			24.2±12.1		

Services	29.4±16.8			24.1±8.8		
Not Specified	33.0±15.6			24.0±9.7		
**Referred trauma**						
Work-related issues	31.8±16.8	3.26	**.02§**	21.1±9.9	1.92	.13
Overall pandemic	31.1±16.2			23.4±9.6		
Threat to family	35.1±16.1			24.5±9.4		
Threat to self	29.0±17.1			23.3±10.1		
**Infected family members**						
No	31.0±16.7	.60	.06	23.4±9.1	-1.00	.32
Yes	33.7±16.0			23.6±10.5		
**Deceased family members**						
No	31.6±16.4	-1.71	.09	23.6±9.4	.19	.85
Yes	35.9±16.5			22.9±11.6		
**Separation from cohabiting family**						
No	30.9±16.6	-3.92	**<.001**	23.4±9.7	-.71	.48
Yes	37.6±14.7			24.2±9.3		
**Separation from non-cohabiting family**						
No	27.9±16.5	-1.55	.12	20.2±8.1	-2.13	**.03**
Yes	32.3±16.5			23.7±9.7		
**Increased workload**						
No	30.2±15.6	-2.60	**.01**	22.9±9.8	-.99	.33
Yes	33.6±17.0			24±9.5		
**Change in habitual tasks**						
No	31.4±16.5	-1.17	.24	23.5±9.6	-.14	.89
Yes	33.2±16.5			23.6±9.9		
**Relocation to other units**						
No	31.3±16.5	-2.04	**.04**	23.5±9.6	-.06	.95
Yes	34.6±16.2			23.5±10.0		
**Unusual exposure to sufferance**						
No	27.3±15.3	-5.80	**<.001**	23.3±9.4	-.34	.74
Yes	35.3±16.5			23.6±9.8		

[1]Impact of Event Scale-Revised; [2]Post Traumatic Growth Inventory - Short Form; * nurse>physician (p=.04); § threat to family>threat to self (p=.02)
